# Vertical transmission of Indian Ocean Lineage of chikungunya virus in *Aedes aegypti* and *Aedes albopictus* mosquitoes

**DOI:** 10.1186/s13071-016-1505-6

**Published:** 2016-04-23

**Authors:** Jakkrawarn Chompoosri, Usavadee Thavara, Apiwat Tawatsin, Rungfar Boonserm, Atchara Phumee, Somchai Sangkitporn, Padet Siriyasatien

**Affiliations:** Department of Medical Sciences, National Institute of Health, Nonthaburi, Thailand; Department of Parasitology, Faculty of Medicine, Chulalongkorn University, Bangkok, Thailand; Excellence Center for Emerging Infectious Diseases, King Chulalongkorn Memorial Hospital, Thai Red Cross Society, Bangkok, Thailand

**Keywords:** Chikungunya virus, Indian Ocean Lineage, *Aedes aegypti*, *Aedes albopictus*, Vertical transmission

## Abstract

**Background:**

The re-emergence of chikungunya (CHIK) fever in Thailand has been caused by a novel lineage of chikungunya virus (CHIKV) termed the Indian Ocean Lineage (IOL). The *Aedes albopictus* mosquito is thought to be a primary vector of CHIK fever in Thailand, whereas *Ae. aegypti* acts as a secondary vector of the virus. The vertical transmission is believed to be a primary means to maintain CHIKV in nature and may be associated with an increased risk of outbreak. Therefore, the goal of this study was to analyze the potential of these two Thai mosquito species to transmit the virus vertically and to determine the number of successive mosquito generations for the virus transmission.

**Methods:**

Two-hundred-and-fifty female *Ae. aegypti* and *Ae. albopictus* mosquitoes were artificially fed a mixture of human blood and CHIKV IOL. Mosquito larvae and adults were sampled and screened for CHIKV by one-step qRT-PCR. LLC-MK2 cell line was used to isolate CHIKV in the mosquitoes each generation. The virus isolate was identified by immunocytochemical staining and was confirmed by sequencing. Both mosquito species fed on human blood without CHIKV and uninfected LLC-MK2 cells were used as controls.

**Results:**

*Aedes aegypti* and *Ae. albopictus* mosquitoes were able to transmit CHIKV vertically to F5 and F6 progenies, respectively. The virus isolated from the two mosquito species caused cytopathic effect in LLC-MK2 cells by 2 days post-infection and immunocytochemical staining showed the reaction between CHIKV IOL antigen and specific monoclonal antibody in the infected cells. DNA sequence confirmed the virus transmitted vertically as CHIKV IOL with E1-A226V mutation. No CHIKV infection was observed in both mosquito species and LLC-MK2 cells from control groups.

**Conclusions:**

The study demonstrated that *Ae. aegypti* and *Ae. albopictus* mosquitoes from Thailand are capable of transmitting CHIKV IOL vertically in the laboratory. Our results showed that *Ae. albopictus* is more susceptible and has a greater ability to transmit the virus vertically than *Ae. aegypti*. This knowledge would be useful for risk assessments of the maintenance of CHIKV in nature, which is crucial for disease surveillance, vector control and the prevention of potential CHIKV epidemics.

## Background

Chikungunya virus (CHIKV) is a mosquito-borne *Alphavirus* belonging to the family Togaviridae. CHIKV is an enveloped, single-stranded, positive-sense RNA virus transmitted to humans through the bite of *Aedes* spp. mosquitoes. The virus was first isolated from humans and mosquitoes in Tanzania, East Africa during the 1952–1953 epidemic [1] and is endemic in countries in Africa and Asia. However, the transmission cycles on these continents are considerably distinct. In Africa, CHIKV is primarily maintained in a sylvatic, enzootic cycle, which involves non-human primates as reservoirs/amplifying hosts and arboreal, primatophilic *Aedes* mosquito species as vectors (*Ae. furcifer-taylori*, *Ae. africanus*, *Ae. luteocephalus*, *Ae. neoafricanus*) [2]. In contrast, CHIKV maintenance in Asia is largely through an endemic/epidemic cycle, in which humans serve as the primary hosts with *Ae. aegypti* historically serving as the primary vector [3]. CHIKV has been previously documented to cause outbreaks with three distinct genotypes based on the E1 envelope glycoprotein sequences: the West African genotype, the East, Central and South African (ECSA) genotypes, and the Asian genotype [4].

An outbreak of chikungunya (CHIK) that increased concern began in coastal Kenya in 2004 [5]. Outbreaks subsequently spread to La Reunion Island and were disseminated rapidly to several countries in the Indian Ocean and India [6, 7], Europe [8, 9], the Americas [10] and Asia [11–13]. CHIKV isolated during the 2005–2006 India Ocean epidemic was a novel ECSA with a mutation from alanine to valine at position 226 in the E1 envelope glycoprotein gene (E1-A226V) and was subsequently described as the Indian Ocean Lineage (IOL) [14, 15]. There are therefore currently four lineages of CHIKV with distinct genotypic and antigenic characteristics [16]. Notably, the amino acid substitution in the E1 gene increases the susceptibility of the *Ae. albopictus* salivary gland to infection and thus enhances the capability of these mosquitoes to transmit the virus to another host [14, 17]. Additionally, viral particles can reach the salivary glands on day 2 post-infection [18], and a high number of viral particles have been detected in eggs on day 6 post-infection [17]. The emergence of this new lineage of CHIKV has resulted in changes in the epidemiological pattern of this disease, with increases in infectivity, vector transmission efficiency, severity and morbidity. This novel lineage of CHIKV has disseminated rapidly, affecting over 60 countries worldwide [12].

In Thailand, a CHIK outbreak was first reported in 1958 [19] with CHIKV of the Asian lineage and *Ae. aegypti* acting as the competent vector [16]. CHIKV had virtually disappeared for 13 years before it resurfaced in the south near the border with Malaysia in 2008 with 200 cases reported by The Department of Disease Control, Ministry of Public Health (MOPH) [20]. A large CHIK outbreak was documented in 2009 with 52,057 cases [21]. After this resurgence, the epidemic has spread across the country, and 76 provinces were affected with the virus in 2012 [22]. Recently, Bueng Kan Province in the northeast reported that 51 out of 109 serum samples showed signs of CHIKV infection [23]. However, CHIKV isolated since 2008 has been classified as IOL with E1-A226V and *Ae. albopictus* has played a major role as a vector [23, 24]. Until now, the CHIK epidemic has principally circulated in the southern provinces where *Ae. albopictus* is abundant.

Vertical or transovarial transmission of virus by mosquito vectors is a mechanism in which infective female mosquitoes are able to pass on viruses to their offspring through their eggs [25]. The transmission is believed to be a primary means by which some arboviruses are kept during adverse environmental conditions. In this duration, the mosquitoes are either inactive or unable to survive but their eggs, particularly the eggs of *Aedes* mosquitoes, are resistant to desiccation and remain viable for longer periods of time, resulting in the possibility of CHIKV retention in eggs [26]. This phenomenon may maintain the viability of viruses during the dry season and the winter when mosquito populations are low [27]. Previous work showed that CHIKV was detected in field-caught male *Ae. albopictus* and *Ae. aegypti* mosquitoes [28–30]. These studies demonstrated the feasibility of virus maintenance through vertical transmission.

In the published literature, several studies have tried to investigate the vertical transmission of CHIKV in the laboratory by using different genotypes, viral titers and *Aedes* spp. [31–34]. Previous reports revealed a failure to prove the experimental vertical transmission of CHIKV IOL in *Ae. albopictus* [32]. Although recent research has shown some evidence of transovarial transmission of CHIKV IOL in experimentally infected *Ae. aegypti* [34], there have not yet been data on the number of successive mosquito generations required for vertical transmission. Therefore, we attempted to demonstrate the vertical transmission of CHIK IOL and the number of successive generations in *Ae. aegypti* and *Ae. albopictus* mosquitoes under laboratory conditions for the risk assessment of CHIKV maintenance in Thailand.

## Methods

### Mosquitoes

*Aedes aegypti* and *Ae. albopictus* mosquitoes used in the experiments were maintained for 3 years at The Biology and Ecology laboratory, National Institute of Health (NIH), Department of Medical Sciences, Nonthaburi Province, Thailand. These two mosquito species were originally obtained from the eggs laid in control ovitraps of the evaluation of lethal ovitraps in Krabi Province in the south of Thailand. Both mosquito species were free of dengue virus and chikungunya virus due to no viruses being detected in their parents prior to the experiment. The mosquitoes were reared in an insectary at 25 ± 3 °C, with 70 ± 20 % relative humidity and a 12:12 light: dark photoperiod. Adult mosquitoes were provided with a mixture of 5 % sucrose and 5 % vitamin B complex soaked in cotton pads. First and second instar larvae were fed 10 mg of thoroughly ground larval food per day, whereas third and fourth instar larvae were fed 65 mg of partially ground larval food per day in filtered water-contained in a plastic tray.

### CHIKV propagation and determination of viral titer

CHIKV IOL was isolated from *Ae. albopictus* collected in epidemic southern Thailand. The procedure for virus propagation and determination of viral titers followed the methods described by Potiwat et al. [35]. Briefly, *Ae. albopictus* C6/36 mosquito cell culture was maintained at 28 °C for virus propagation. The CHIKV isolated from the infected mosquitoes was propagated in a monolayer of C6/36 cell line at passage level 5. The supernatant was used to determine the viral titer via plaque assay.

### Confirmation of CHIKV

Prior to the experiment, the virus lineage was confirmed by a one-step RT-PCR technique using the two outer primer pairs targeting the E1 gene of CHIKV [E1-10145 F: 5'- ACAAAACCGTCATCCCGTCTC-3' genome position 10145-10165 and E1-11158R: 5'- TGACTATGTGGTCCTTCGGAGG-3' genome position 11137-11158] [36]. The supernatant of CHIKV-infected C6/36 cell line was extracted for viral RNA using Invisorb® Spin Virus RNA Mini Kit (InvitexGmbh, Germany). The RT-PCR technique was performed using BluePrintTM One Step RT-PCR Kit which has a detection sensitivity as low as 0.1 pg of HL60 total RNA as specified in the kit instructions. Target RNA was amplified in a 25-μl volume containing 12.5 μl of 2xOne-Step BluePrint^TM^ Buffer, 1.0 μl of One Step BluePrint^TM^ RT Enzyme Mix, 1.0 μl (10 mM) of each primer, 7.5 μl of nuclease‐free water, and 2.0 μl of RNA template. A thermal cycler using PCR Mastercycler ® Pro (Eppendorf, Hamburg, Germany) was programmed to incubate at 50 °C for 30 min, 95 °C for 15 min followed by 40 cycles of 95 °C for 1 min, 64 °C for 1 min and 72 °C for 1 min, with a final cycle of 72 °C for 10 min and a final holding at 4 °C (PCR cycling conditions adjusted to optimize the reactions and based on the melting temperature of primers and kit instructions). A 6-μl product was electrophoresed through a 1 % agarose gel at 100 V, stained with ethidium bromide, and visualized on a UV transilluminator for positive bands. The PCR products were directly cloned into pGEM-T Easy vector (Promega corporation, USA), which followed the kit instructions. Ligation reactions were transformed into competent bacterial cells for screening transformants (white colonies generally containing inserts). The transformants with inserts confirmed by PCR technique were cultured for making copies of DNA and DNA was then purified for sequencing.

### Vertical transmission experiment

Two-hundred-and-fifty unfed 4- to 5-day-old female *Ae. aegypti* and *Ae. albopictus* mosquitoes per cage (three cages/replicate) were used for artificial blood-feeding. Each mosquito species was divided into 2 groups: one was fed only human blood as a control, whereas the other was provided with the human blood-virus mixture as a treatment. The mosquitoes were allowed to feed for 45 min through a parafilm covering the base of a glass chamber containing either only human blood or the human blood-virus mixture. The mixture was composed of human blood, and the virus at a final concentration of 10^5^ PFU/ml and was maintained at 37 °C during the feeding period. The experiment was carried out in triplicate. Fully engorged females were maintained with 5 % sucrose solution supplemented with 5 % vitamin B complex at 25 ± 3 °C. Two days after the blood-meal, a black plastic bowl containing filtered water and filter paper was placed into the mosquito cages for oviposition. The filter paper with eggs was collected and replaced with the new paper until no oviposition (approximately day 5-6 post-infection). Eggs aged at least 3 days and collected on the different dates were pooled and allowed to hatch in a white plastic tray. The remaining eggs were kept for further use. The larvae were reared to pupae and adults to maintain subsequent progeny. The adults of F1–F9 generations of the experimental group were fed on human blood without CHIKV. Six hundred of the fourth instar larvae and female adults per replicate were randomly collected and gathered into 30 pools/replicate (20 larvae or female adults/pool) for the detection of CHIKV.

### Demonstration of CHIKV in progeny

#### Detection of CHIKV RNA

Each pool of larvae and adults was extracted for viral RNA using Invisorb® Spin Virus RNA Mini Kit (InvitexGmbh, Germany). One-step qRT-PCR was performed using *ab*TES™ DEN 5 qPCR I Kit (AIT biotech Pte. Ltd, Singapore) which has an analytical sensitivity (limit of detection) for detection of CHIKV as low as 0.0043 PFU/ul (4.3 PFU/ml) when used on CFX96 Real-Time System as specified in kit instructions. Target RNA was amplified in a 25-μl volume containing 12.5 μl of 2× RT-PCR reaction mix, 0.5 μl of RT/*Taq* enzyme mix, 1.5 μl of primer/probe mix (the concentration not specified in kit components), 0.1 μl of PCR enhancer template, 5.4 μl of nuclease‐free water, and 5.0 μl of RNA template. A thermal cycler using CFX96 Real-Time System (Bio-Rad Laboratories, Inc, USA) was programmed to incubate at 53 °C for 10 min, 95 °C for 2.5 min and then to proceed with 41 cycles of 95 °C for 17 s, 59 °C for 31 s and 68 °C for 32 s, with a final cycle of 68 °C for 7 min and a final holding at 4 °C (PCR cycling conditions following the kit instructions). CHIK viral RNA extracted from CHIK patients’ serum was used as a positive control, and RNA extracted from uninfected larvae and adults were employed as negative control.

#### CHIKV isolation and propagation

The remaining eggs of each generation of *Ae. aegypti* and *Ae. albopictus* mosquitoes which were positive for CHIKV RNA detection were hatched into larvae to confirm the presence and the viability of CHIKV in the successive generations of mosquitoes. Pools of mosquito larvae were homogenized using a Mixer Mill (Tissuelyser, Qiagen, Germany) in 15 ml sterile plastic tubes and 1.5 ml of MEM containing 2 % fetal bovine serum, 50 units/ml Penicillin and 100 ug/ml Streptomycin (Gibco, Invitrogen, USA). Samples were centrifuged at 12,000 rpm for 20 min at 4 °C. The rhesus monkey kidney continuous cell line, LLC-MK2 was used for virus propagation. The cell line was cultured with Eagle’s minimum essential medium (MEM) (Sigma-Aldrich, USA) with 10 % heat-inactivated fetal bovine serum (FBS) (Gibco, Invitrogen, USA), 100 U/ml penicillin and 100 μg/ml streptomycin (Gibco, Invitrogen, USA), and maintained at 37 °C with 5 % CO_2_. Clarified homogenates were inoculated into 6 well plate (Nunclon, Denmark) spread with a monolayer of LLC-MK2 cells for 1 h at constant temperature. After discarding and refreshing with 2 mL medium, the cell cultures were incubated with the same conditions for 6–7 days. Cytopathic effects (CPE) were checked every 8 h after incubation for 24 h and observation over the next 6–7 days. Two days after infection, the culture supernatants were harvested and cellular debris was removed by centrifugation at 12,000 rpm. The supernatants were stored at -80 °C until identification and sequencing. The uninfected LLC-MK2 cells were used as negative control and the cells inoculated with 10^4^ PFU/ml of CHIKV IOL stock at MOI = 1 were employed as positive control.

#### Demonstration of CHIKV by immunocytochemistry (ICC) staining

CHIKV isolate in LLC-MK2 cell culture was identified by ICC staining. Infected and uninfected cell suspensions were applied to SuperFrost Plus microscope slides (Thermo Scientific, USA), which were then air dried and fixed in 100 % cold acetone and rehydrated in graded alcohols. The slides were stained with primary antibody, mouse monoclonal [B1412huM] (Abcam, Cambridge, MA, USA) to CHIKV and mouse IgG2a antibody (HRP) (Abcam, Cambridge, MA, USA) as the secondary antibody. The color was developed by using DAB (3,3′-diaminobenzidine) and counterstained with hematoxylin (Dako, Carpenteria, CA), which was then examined under a light microscope (Olympus, Japan) at 100× magnification.

#### Determination of minimum infection rate (MIR)

Both larval and adult pools of the two mosquito species positive for CHIKV were determined for the MIR, which was calculated from the number of positive pools divided by the number of larvae or adults tested × 1000 [37] and expressed as ratio (proportion of positive pools by the number of larvae tested).

### Data analysis

Mean CHIKV RNA titers of *Ae. aegypti* and *Ae. albopictus* larvae and adults were analyzed and compared between two groups within the species by unpaired t-test, among generations within the species and among four groups from the two mosquito species within the generation by one-way ANOVA and Bonferroni’s multiple comparison test with a significance level of 0.05 using GraphPad Prism 5.0 software.

## Results

### Vertical transmission rates in *Ae. aegypti* and *Ae. albopictus*

**Fig. 1:**
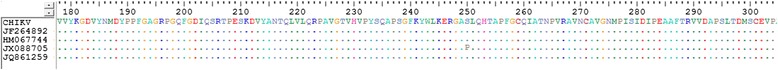
An alignment of amino acid sequence of the E1 gene of CHIKV IOL isolate from the South of Thailand. The sequence showed the position of the A226V mutation indicated by a vertical column

CHIKV IOL with the A226V mutation in E1 gene used in this experiment was confirmed by sequencing (Fig. 1). Total RNA extracted from each pool of *Ae. aegypti* and *Ae. albopictus* fourth instar larvae and adults of both infected and uninfected mosquitoes each generation (F) was measured for CHIKV using the one-step qRT-PCR technique. The experiment revealed that CHIKV IOL was detectable until the F5 generation of *Ae. aegypti* larvae and adults, whereas the persistence of the virus in *Ae. albopictus* larvae and adults was found to be to the F6 generation. Mean MIRs with standard deviation (SD) were achieved for both mosquito species (Table 1). In *Ae. aegypti*, mean MIRs with SD determined were 8.33 ± 2.887, 3.33 ± 1.665, 5.00 ± 1.670, 3.33 ± 0.000 and 1.67 ± 0.000 for larvae and 10.00 ± 3.333, 6.67 ± 3.335, 5.00 ± 3.333, 3.33 ± 1667 and 1.67 ± 1.667 for adults from the F1, F2, F3, F4 and F5 generations, respectively, whereas the virus was absent at the F6-F9 generations. MIRs were also expressed as a ratio. Thus, the ratios measured were 1:120, 1:300, 1:200, 1:300, and 1:600 for *Ae. aegypti* larvae and 1:100, 1:150, 1:200, 1:300 and 1:600 for *Ae. aegypti* adults from the F1, F2, F3, F4 and F5 generations, respectively. For *Ae. albopictus*, mean MIRs with SD determined were 18.33 ± 3.333, 8.33 ± 2.887, 6.67 ± 1.667, 3.33 ± 1.667, 5.00 ± 0.000 and 1.67 ± 0.000 for larvae and 21.67 ± 2.887, 11.67 ± 1.667, 8.33 ± 1.667, 6.67 ± 1.667, 5.00 ± 1.667 and 1.67 ± 1.667 for adults from the F1, F2, F3, F4, F5 and F6 generations, respectively, whereas the virus was undetectable at the F7-F9 generations (Table 1). The ratios also calculated were 1:54.5, 1:120, 1:150, 1:300, 1:200, and 1:600 for *Ae. albopictus* larvae and 1:46.2, 1:85.7, 1:120, 1:150, 1:200 and 1:600 for *Ae. albopictus* adults from the F1, F2, F3, F4, F5 and F6 generations, respectively. However, CHIKV was not detected from *Ae. aegypti* and *Ae. albopictus* larvae and adults from the control groups.

**Table 1:** Minimum infection rate (MIR) of *Ae. aegypti* and *Ae. albopictus* through successive generations

		*Ae. aegypti*						*Ae. albopictus*					
Generation	No. tested	Larvae			Adults			Larvae			Adults		
		Positive pools/Tested pools	MIR (Mean ± SD)	Ratio	Positive pools/Tested pools	MIR (Mean ± SD)	Ratio	Positive pools/Tested pools	MIR (Mean ± SD)	Ratio	Positive pools/Tested pools	MIR (Mean ± SD)	Ratio
F1	600	5/30	8.33 ± 2.887	1:120	6/30	10.00 ± 3.333	1:100	11/30	18.33 ± 3.333	1:54.5	13/30	21.67 ± 2.887	1:46.2
F2	600	2/30	3.33 ± 1.665	1:300	4/30	6.67 ± 3.335	1:150	5/30	8.33 ± 2.887	1:120	7/30	11.67 ± 1.667	1:85.7
F3	600	3/30	5.00 ± 1.670	1:200	3/30	5.00 ± 3.333	1:200	4/30	6.67 ± 1.667	1:150	5/30	8.33 ± 1.667	1:120
F4	600	2/30	3.33 ± 0.000	1:300	2/30	3.33 ± 1.667	1:300	2/30	3.33 ± 1.667	1:300	4/30	6.67 ± 1.667	1:150
F5	600	1/30	1.67 ± 0.000	1:600	1/30	1.67 ± 1.667	1:600	3/30	5.00 ± 0.000	1:200	3/30	5.00 ± 1.667	1:200
F6	600	0/30	0	0	0	0	0	1/30	1.67 ± 0.000	1:600	1/30	1.67 ± 1.667	1:600
F7	600	0/30	0	0	0	0	0	0/30	0	0	0	0	0
F8	600	0/30	0	0	0	0	0	0/30	0	0	0	0	0
F9	600	0/30	0	0	0	0	0	0/30	0	0	0	0	0

Each value is the average of the three independent experiments

Ratio: Proportion of positive pool(s) by no. of larvae tested

MIR expressed as Ratio

### Quantification of CHIKV in *Ae. aegypti* and *Ae. albopictus*

**Fig. 2:**
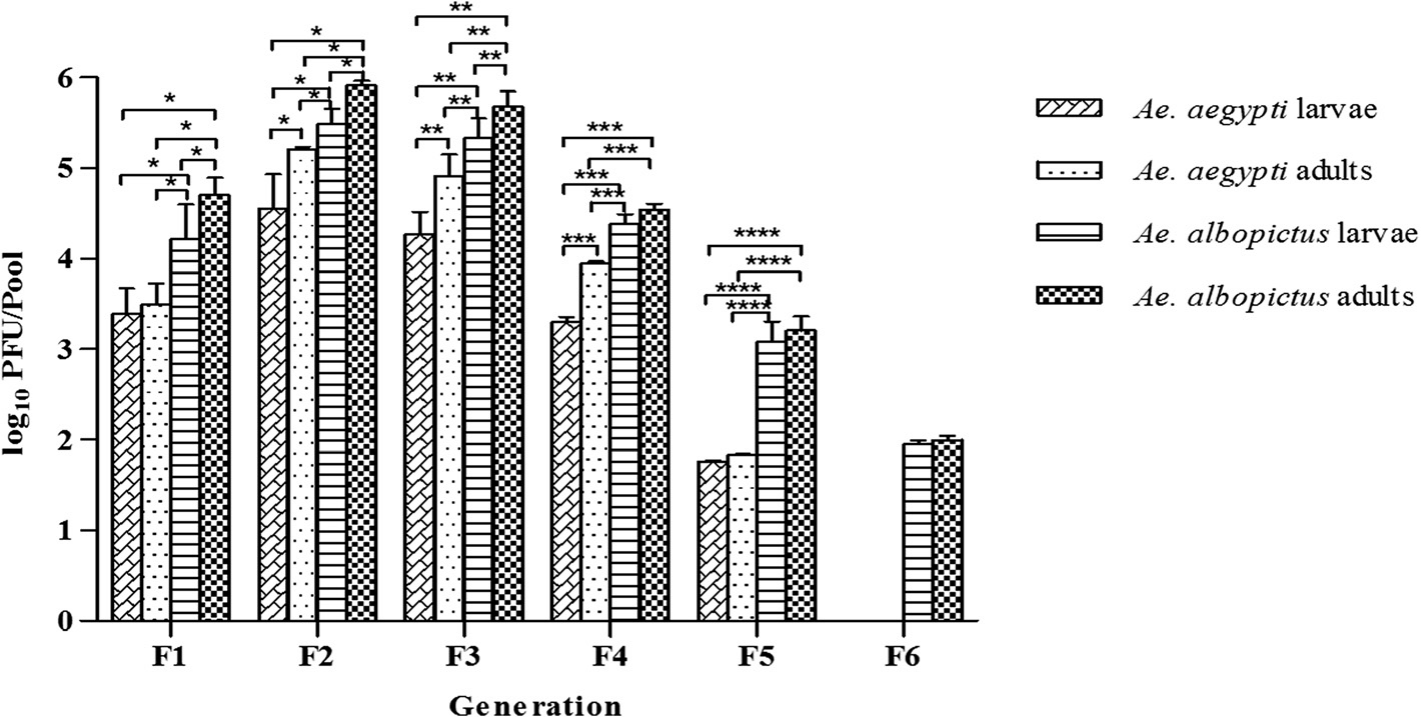
Vertical transmission of Chikungunya virus in *Ae. aegypti* and *Ae. albopictus* as determined by measuring log_10_ PFU/pool of larvae and adults by real time RT-PCR. The graph shows mean value of viral titer (PFU/pool) with standard deviation of larvae and adults of both mosquito species each generation. No significant difference was observed among the generations of *Ae. aegypti* larvae (*P* = 0.2223) but there were significant differences observed among the generations of *Ae. aegypti* adults (*P* < 0.0001), *Ae. albopictus* larvae (*P* < 0.0001) and *Ae. albopictus* adults (*P* < 0.0001). Significant differences were also observed among those four groups within the generation in the F1, F2, F3, F4, and F5 generations. Asterisks are indicating significant difference among those groups (*P < 0.0001, **P = 0.0055, ***P = 0.0009, and ****P = 0.0141)

The CHIKV RNA titers as determined by qRT-PCR in each generation of *Ae. aegypti* and *Ae. albopictus* were measured by log_10_ as shown in Fig. 2. In *Ae. aegypti*, the viral RNA titers measured were 10^2.5^ to 10^3.8^ (10^3.3^ ± 10^0.6^) PFU/pool, 10^3.2^ to 10^4.8^ (10^4.5^ ± 10^1.2^) PFU/pool, 10^3.2^ to 10^4.5^ (10^4.2^ ± 10^0.4^) PFU/pool, 10^3.2^ to 10^3.3^ (10^3.3^ ± 10^0.1^) PFU/pool, and 10^1.7^ to 10^1.8^ (10^1.8^ ± 10^0.01^) PFU/pool for larvae and 10^2.5^ to 10^3.8^ (10^3.4^ ± 10^0.5^) PFU/pool, 10^5.2^ to 10^5.3^ (10^5.2^ ± 10^0.03^) PFU/pool, 10^4^ to 10^5.2^ (10^4.8^ ± 10^0.6^) PFU/pool, 10^3.9^ to 10^4.1^ (10^4.0^ ± 10^0.01^) PFU/pool, and 10^1.8^ to 10^1.9^ (10^1.8^ ± 10^0.02^) PFU/pool for adults from the F1, F2, F3, F4 and F5 generations, respectively. The viral RNA titers were compared among the generations of *Ae. aegypti*. No significant difference was observed among the generations of *Ae. aegypti* larvae (*P* = 0.2223). However, there was significant difference among the generations of *Ae. aegypti* adults as follows: F1vs F2, F1 vs F3, F2 vs F3, F2 vs F4, F2 vs F5 and F3 vs F5 (*P* < 0.0001). For *Ae. albopictus*, the viral RNA titers measured were 10^3.7^ to 10^4.9^ (10^4.2^ ± 10^1.2^) PFU/pool, 10^5.0^ to 10^5.6^ (10^5.4^ ± 10^0.3^) PFU/pool, 10^4.6^ to 10^5.6^ (10^5.2^ ± 10^0.4^) PFU/pool, 10^4.3^ to 10^4.5^ (10^4.4^ ± 10^0.1^) PFU/pool, 10^2.6^ to 10^3.3^ (10^3.0^ ± 10^0.4^) PFU/pool and 10^1.9^ to 10^2.0^ (10^1.9^ ± 10^0.04^) PFU/pool for larvae and 10^4.0^ to 10^5.0^ (10^4.7^ ± 10^0.3^) PFU/pool, 10^5.8^ to 10^6.0^ (10^5.9^ ± 10^0.1^) PFU/pool, 10^5.2^ to 10^5.9^ (10^5.6^ ± 10^0.3^) PFU/pool, 10^4.5^ to 10^4.6^ (10^4.5^ ± 10^0.1^) PFU/pool, 10^3.1^ to 10^3.4^ (10^3.2^ ± 10^0.2^) PFU/pool and 10^1.9^ to 10^2.0^ (10^1.9^ ± 10^0.05^) PFU/pool for adults from the F1, F2, F3, F4, F5 and F6 generations, respectively. The viral RNA titers were compared among the generations of *Ae. albopictus* as well. Significant differences were observed among the generations of *Ae. albopictus* larvae as follows: F1 vs F2, F1 vs F3, F2 vs F4, F2 vs F5, F2 vs F6, F3 vs F5 and F3 vs F6 (*P* < 0.0001) and among the generations of *Ae. albopictus* adults as follows: F1 vs F2, F1 vs F3, F2 vs F3, F2 vs F4, F2 vs F5, F2 vs F6, F3 vs F4, F3 vs F5 and F3 vs F6 (*P* < 0.0001). The CHIKV RNA titers were also compared among four groups within generation: *Ae. aegypti* larvae, *Ae. aegypti* adults*, Ae. albopictus* larvae and *Ae. albopictus* adults. Significant differences were observed among those groups in the F1 (*P* < 0.0001), F2 (*P* < 0.0001), F3 (*P* = 0.0055), F4 (*P* = 0.0009) and F5 (*P* = 0.0141) generations as shown in Fig. 2. For the F6 generation, the CHIKV RNA titers were compared between *Ae. albopictus* larvae and *Ae. albopictus* adults, but no significant difference was observed between the two groups (*P* = 0.2899). All CHIKV RNA titers determined from 5 generations of *Ae. aegypti* larvae and those of *Ae. aegypti* adults were compared and the significant difference was observed between the two groups (*P* = 0.0026). Likewise, all CHIKV RNA titers measured from 6 generations of *Ae. albopictus* larvae and those of *Ae. albopictus* adults were compared and the significant difference was observed between the two groups (*P* = 0.0003). The results indicated that the RNA titers determined in both *Ae. aegypti* and *Ae. albopictus* larvae and adults peaked at the F2 generation and declined until no detection of the virus in the F6–F9 generations of *Ae. aegypti* and the F7–F9 generations of *Ae. albopictus*.

### CHIKV propagation in LLC-MK2 cells

**Fig. 3:**
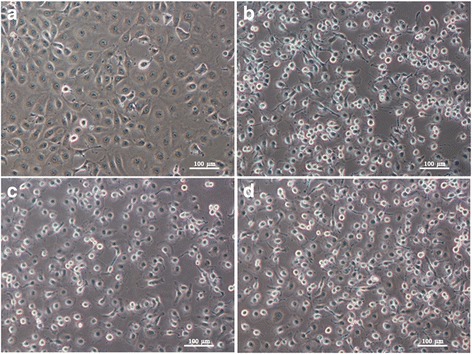
The CHIKV isolation in LLC-MK2 cells. The CPE in infected cells was produced 2 days after CHIKV inoculation. The infected cells was rounded and fused with the adjacent cells for syncytia formation. **a** Monolayer of uninfected cells (negative control); **b** The infected cells after inoculation with CHIKV stock (positive control); **c** The infected cells after inoculation with homogenate from *Ae. aegypti* larvae; **d** The infected cells after inoculation with homogenate from *Ae. albopictus* larvae. (10× magnification)

*Aedes aegypti* and *Ae. albopictus* larvae of each generation which were positive for CHIKV were collected and processed for virus propagation. A virus isolate was identified by CPE in LLC-MK2 cells which included rounding of the infected cells, fusion with adjacent cells to form syncytia by day 2 post-infection (Fig. 3). The CPE characteristic was observed in LLC-MK2 cells inoculated with the homogenate from the F1 to F4 generations of *Ae. aegypti* (Fig. 3c) and from the F1 to F5 generations of *Ae. albopictus* (Fig. 3d). No CPE appeared in the cells inoculated with homogenate from the F5 generation of *Ae. aegypti* and from the F6 generation of *Ae. albopictus*, even though their larvae and adults were found positive for CHIKV by qRT-PCR. The LLC-MK2 cells inoculated with CHIKV IOL stock showed the CPE aspects in all batches of experiments (Fig. 3b), whereas no CPE was observed in LLC-MK2 cells without the CHIKV inoculation (Fig. 3a).

### Determination of CHIKV in LLC-MK2 cells

**Fig. 4:**
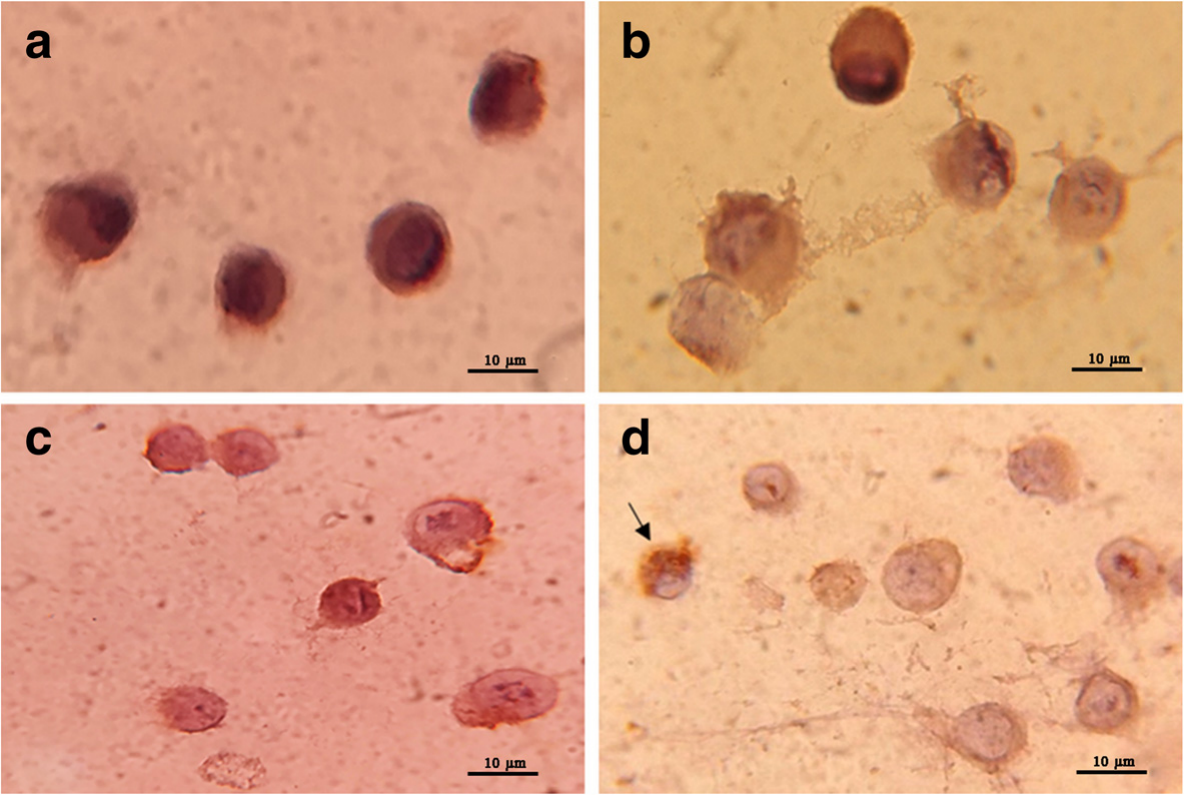
Immunocytochemical (ICC) staining of CHIKV IOL in the infected LLC-MK2 cells using anti-chikungunya virus antibody. The *arrow* indicates CHIKV IOL antigen with dark brown within the cytoplasm of the infected cells). **a** Uninfected LLC-MK2 cells with blue staining (negative control); **b** CHIKV IOL antigen in the infected LLC-MK2 inoculated with CHIKV IOL stock; **c** CHIKV IOL antigen in the infected LLC-MK2 cells inoculated with homogenate from *Ae. aegypti* larvae; **d** CHIKV IOL antigen in the infected LLC-MK2 cells inoculated with homogenate from *Ae. albopictus* larvae. (100 × magnification)

Apart from demonstration of CHIKV propagations by the CPE in LLC-MK2 cells. The virus antigen was also detected using an immunocytochemical technique (Fig. 4). The positive staining for CHIKV IOL showed distinct brown caused by oxidation of DAB by HRP within the cytoplasm of LLC-MK2 infected with the CHIKV IOL stock (Fig. 4b), the CHIKV IOL isolated from *Ae. aegypti* (Fig. 4c) and the CHIKV IOL isolated from *Ae. albopictus* (Fig. 4d). However, the negative staining for CHIKV IOL remained blue in the uninfected cells (Fig. 4a). This showed that the reaction between specific monoclonal antibody and CHIKV IOL antigen isolated from *Ae. aegypti* and *Ae. albopictus* has given satisfactory results.

### RT-PCR analysis and DNA sequencing

**Fig. 5:**
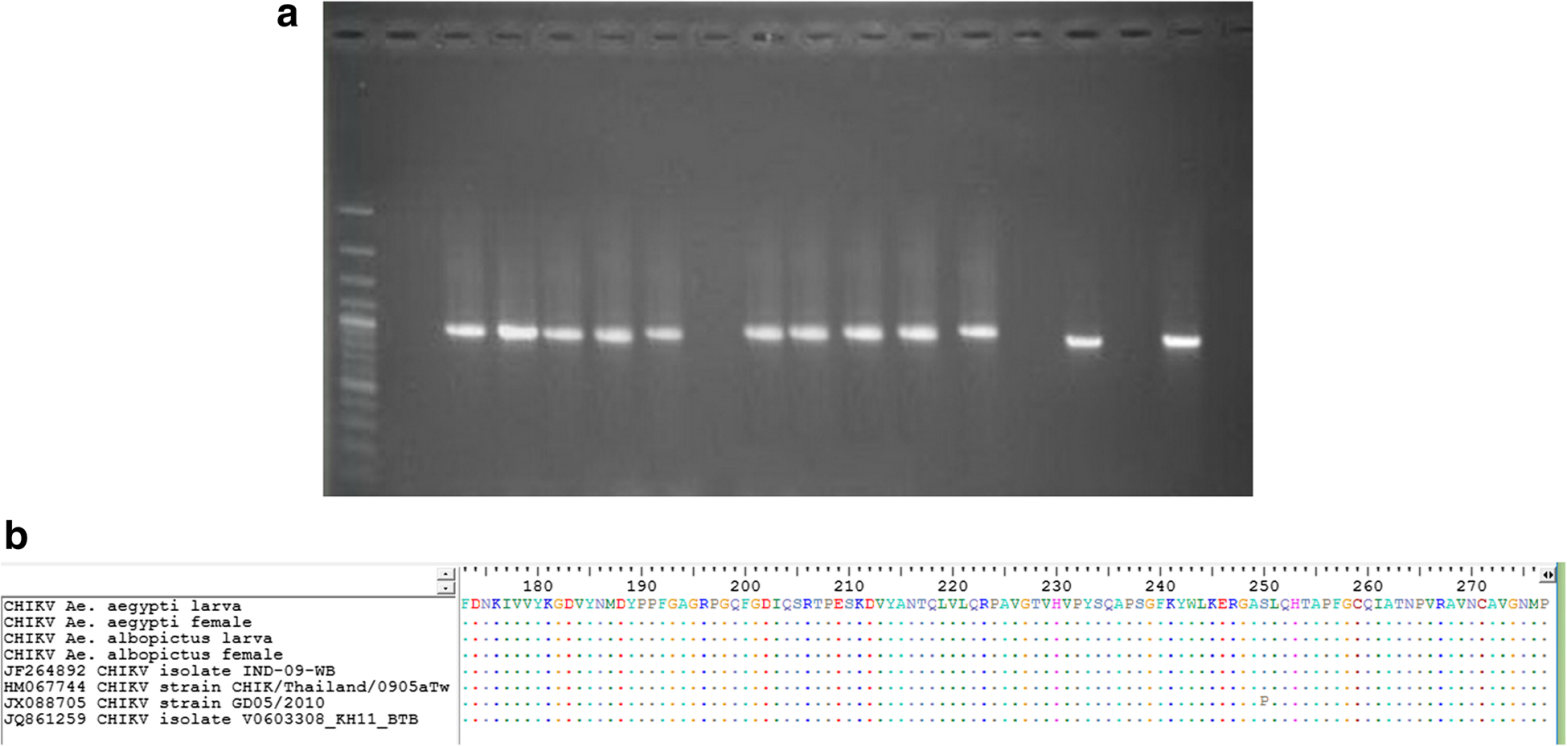
Agarose gel analysis of the PCR products generated by RT-PCR assay and alignment of the amino acid sequence of CHIKV. **a**. 1014 bp PCR products were reverse-transcribed from CHIKV isolated from *Ae. aegypti* and *Ae. albopictus*, M: DNA marker, Lane 1: the uninfected LLC-MK2 cells (negative control), Lane 2: the CHIKV stock-infected LLC-MK2 cells (positive control), Lanes 3–6: the infected LLC-MK2 cells from the F1–F4 *Ae. aegypti* larvae, Lane 7: the uninfected LLC-MK2 cells from the F5 *Ae. aegypti* larvae, Lanes 8–12: the infected LLC-MK2 cells from the F1–F5 *Ae. albopictus* larvae, Lane 13: the uninfected LLC-MK2 cells from the F6 *Ae. albopictus* larvae, Lanes 14–15: the RNA samples from the F4–F5 *Ae. aegypti* adults positive for CHIKV by qRT-PCR, Lanes 16–17: the RNA samples from the F5–F6 *Ae. albopictus* adults positive for CHIKV by qRT-PCR, respectively; **b**. An alignment of amino acid sequences confirmed the virus transmitted vertically in *Ae. aegypti* and *Ae. albopictus* as CHIKV IOL with the A226V mutation in E1 gene as indicated by a vertical column

The supernatants of LLC-MK2 cells inoculated with homogenate from the F1–F5 *Ae. aegypti* larvae and the F1–F6 *Ae. albopictus* larvae which were kept at -80 °C including the remaining RNA extracted from the F4–F5 *Ae. aegypti* adults and the F5–F6 *Ae. albopictus* adults after performing qRT-PCR were further processed using a one-step RT-PCR technique. A 1014 bp-PCR product was indicative of a positive band for CHIKV, which was analyzed in the RNA samples from the F1, F2, F3, and F4 generations of *Ae. aegypti* and the RNA samples from the F1, F2, F3, F4 and F5 generations of *Ae. albopictus*. Nevertheless, no band pattern specific for CHIKV appeared from the RNA samples from the F5 generation of *Ae. aegypti* and the F6 generation of *Ae. albopictus* (Fig. 5a). After the cloning of PCR product into the vector, bacterial transformation, screening by PCR technique, DNA purification, and DNA sequencing, all DNA sequences obtained from those generations of the two mosquito species were converted into amino acid sequences that confirmed the virus transmitted vertically as CHIKV IOL with the A226V mutation in E1 gene (Fig. 5b).

## Discussion

Arboviruses are maintained by biological transmission, which depends on host and vector coexistence in time and space. Alternative transmission contributing to the preservation of a virus in nature is vertical transmission, which tends to show a low transmission rate but is of increased importance in endemic areas as an overwinter mechanism. The vertical transmission of arboviruses has been demonstrated in field-caught mosquitoes [38–40] and has also been shown in laboratory experiments [41–44]. Dengue virus (DENV) in the genus *Flavivirus* has been found in male *Ae. aegypti* and *Ae. albopictus* mosquitoes collected in the field [45–47]. Previous studies showed that DENV was transmittable transovarially in *Ae. aegypti* [48, 49] and was isolated from *Ae. aegypti* larvae until the F5 generation, which was the confirmatory evidence of vertical transmission [49]. DENV was also detected in *Ae. albopictus* progenies where parental females were infected by parenteral inoculation of virus [50]. Moreover, the dengue infection rate increased in the successive generations of *Ae. albopictus* [51]. Ross River virus belonging to the *Alphavirus* group was present in wild-caught male *Aedes* mosquitoes, providing a source of natural vertical transmission of this group of viruses [52]. In addition, the viruses in the same group, namely, Sindbis virus and Western equine encephalomyelitis virus, were found in adult *Aedes* species developed from larvae that were collected from natural habitats as well [53, 54].

It has long been in doubt whether the CHIKV is maintained in nature by the aforementioned mechanism. So far, researchers have made an effort to demonstrate the experimental vertical transmission of CHIKV in several species of *Aedes* mosquitoes, however, they failed to isolate the virus in *Ae. aegypti formosus* and *Ae. furcifer* from South Africa [33] and in *Ae. aegypti* and *Ae. albopictus* from India [31]. Even more worrisome, data on vertical transmission of CHIKV under laboratory conditions have been scarce and inconsistent. Concerns over this phenomenon were raised when CHIKV was detected in male *Ae. aegypti* and *Ae. albopictus* mosquitoes caught in epidemic areas [28, 30]. Hence, Vazeille and others tried to conduct vertical transmission experiments using CHIKV IOL and *Ae. albopictus* strains from La Reunion, but they were unable to isolate the virus from the mosquito [32]. Until recently, previous studies have elucidated the existence of a transovarial transmission pattern of this novel virus in *Ae. aegypti* larvae and adults [34]. Although these studies showed evidence of experimental vertical transmission of the new lineage of CHIKV, there has not yet been information on the number of mosquito generations in which the virus persists. Our study demonstrated that the virus can be transmitted transovarially under laboratory conditions up to the F5 and the F6 successive generations of *Ae. aegypti* and *Ae. albopictus*, respectively. The present work also indicated that the CHIKV IOL was detectable in *Ae. aegypti* and *Ae. albopictus* larvae and adults developed from eggs in the first gonotrophic cycle which their parental females were provided with infectious blood meal. Our result, in contrast to the earlier report showing that the *Ae. aegypti* larvae and adults developed directly from the collected eggs within 2–3 days following the infectious blood meal were found negative for CHIKV RNA [34]. This failure was primarily due to a shorter gonotrophic cycle and the inadequate dissemination of the virus to ovaries and oviduct. In addition, several factors might be associated with this negative result including the low number of field-caught mosquitoes tested, poor efficiency of blood-feeding of the nature collected-mosquitoes which caused very low level of virus infection, egg production and oviposition rate. However, our experiment used 250 female adults of both *Ae. aegypti* and *Ae. albopictus* per cage from laboratory colonies without CHIKV IOL for infectious blood feeding. The use of CHIKV IOL-free mosquitoes is to ensure that the virus infection in both mosquito species occurred from our experiment. As determined by visual inspection, most of the mosquitoes in the cage were fully engorged and laid all their eggs by 5–6 days post-infection. The later oviposition provided a chance to obtain the eggs infected with CHIKV IOL which is in accordance with the previous study revealing that the *Ae. albopictus* eggs became infected with the virus at day 6 post-infection [17].

The mean MIRs determined in this study were highest in the F1 generation of *Ae. aegypti* (8.33 ± 2.887, 1:120 for larvae and 10.00 ± 3.333, 1:100 for adults) and *Ae. albopictus* (18.33 ± 3.333, 1:54.5 for larvae and 21.67 ± 2.887, 1:46.2 for adults). Such high MIRs in that generation were similar to the preceding evidence that showed the highest MIR in the F1 generation of *Ae. aegypti* infected with DENV 2 [49]. This high MIR could be used as an indicator of potential outbreaks. Our result also revealed that the MIRs determined in early generations of *Ae. aegypti* and *Ae. albopictus* adults were higher than those measured in their larvae. It was due to the amplification of virus following transition from larvae to adult stage [34]. However, there was fluctuation of MIR observed in larvae from the F3 generation of *Ae. aegypti* and the F5 generation of *Ae. albopictus*. This was feasible because of the different number of eggs obtained each generation of the mosquitoes and the random sampling of progeny from virus-positive mosquitoes which might result in a minor variation in MIR of those generations. Nevertheless, the CHIKV RNA titer measured in the F1 generation of *Ae. aegypti* and *Ae. albopictus* was low, peaked at the F2 generation and decreased subsequently to be undetectable to the F6–F9 generations and the F7–F9 generations of *Ae. aegypti* and *Ae. albopictus*, respectively. A low amount of CHIKV RNA titer determined in the larvae and adults of *Ae. aegypti* and *Ae. albopictus* from the F1 generation was due to low viral infection in eggs that the most eggs were laid prior to day 6 post-infection. Furthermore, the adaptation between the virus and the mosquitoes may be associated with such low CHIKV RNA titer from the initial generation. The viral titer increased up to the F2 generation, however, it decreased subsequently in the later generations of the two mosquito species that may be involved with the genetic factors of both virus and mosquitoes including the selection pressure under laboratory conditions. The present study showed that the viral RNA titer was not significantly different among generations of *Ae. aegypti* larvae, whereas it revealed a significant difference among generations of *Ae. aegypti* adults, *Ae. albopictus* larvae and *Ae. albopictus* adults. The viral RNA titers determined in *Ae. aegypti* adults and *Ae. albopictus* adults were significantly higher than those measured in *Ae. aegypti* larvae and *Ae. albopictus* larvae, respectively. It showed that the amplification of CHIKV occurred after the larval stage [34]. In addition, the viral RNA titers determined in the F1–F5 generations of *Ae. albopictus* were significantly higher than those measured in the same generations of *Ae. aegypti*. This result supported the previous work reporting that the novel lineage of CHIKV was well-adapted to *Ae. albopictus* [14, 17, 18]. The viability of CHIKV IOL transmitted by both mosquito species from generation to generation was confirmed by the marked CPE in LLC-MK2 cells inoculated with the homogenates of *Ae. aegypti* adults from the F1–F4 generations and that of *Ae. albopictus* adults from the F1–F5 generations. This characteristic could be generated within 6 to 7 days post-infection and the CHIKV IOL antigen was also visualized in the cells by ICC staining. However, no CPE was observed within the cells tested with the homogenates from the last generation of the two mosquito species positive for CHIKV. It might happen as a result of a very low viral titer determined in those mosquito generations. The virus isolated from the F1–F4 *Ae. aegypti* and the F1–F5 *Ae. albopictus* larvae including the RNA samples extracted from the F4 *Ae. aegypti* and the F5 *Ae. albopictus* adults were also confirmed as CHIKV IOL when the alignment of amino acid sequence showed the position of A226V mutation in E1 gene. Taken together, it elucidated that these two Thai mosquito species were indeed able to transmit CHIKV IOL vertically. In our experiment, the RNA samples from the F5 generation of *Ae. aegypti* and the F6 generation of *Ae. albopictus* found positive for CHIKV by qRT-PCR were negative for the virus by RT-PCR. The possible reason is that the qRT-PCR is more sensitive and reproducible than conventional RT-PCR. It was similar to the previous study showing that the qRT-PCR provided a higher positive result of DENV and CHIKV detection than conventional RT-PCR [55].

In the present experiment, CHIKV IOL was found to be transmitted by the *Ae. aegypti* mosquito, which is also primary vector of DENV in Thailand. This *Aedes* species is more closely associated with human habitats and is commonly found across the country, whereas *Ae. albopictus* is likely to be more plentiful in the South [30]. Our result revealed that *Ae. albopictus* is more competent at CHIKV infection and transmission than *Ae. aegypti*. In contrast, the previous study reported no apparent difference in the capacities of experimental vertical transmission of the two *Aedes* species examined [43]. However, the occurrence of this phenomenon is greatly variable depending on the mosquito species and the virus. The results indicated that both *Ae. aegypti* and *Ae. albopictus* are capable of maintaining CHIKV IOL in nature. But it is because *Ae. albopictus* is a more competent vector with virus infectivity and transmission, the high incidence of CHIK has been observed in the southern provinces of Thailand where the *Ae. albopictus* population is much greater than that of *Ae. aegypti*. Such high incidence of CHIK in the South may also be associated with the environmental factors stimulating the outbreak. The introduction of this virus into the South coinciding with high vector abundance and activity as well as optimal temperature may cause a CHIK outbreak. As reported earlier, outbreak locations had monthly mean temperatures of 20–26 °C [56, 57], so that the temperature could be one of the significant factors for the initiation of CHIK outbreaks. Since this region of Thailand has a longer rainy season than others [58], it provides more breeding sites for *Ae. albopictus*. Furthermore, high precipitation moderates the temperature, which may be suitable for the concurrence of virus-vector-host interactions leading to the large CHIK outbreak in 2009 [21]. Presently, *Ae. albopictus* has invaded all five continents under current climate conditions and acts as a main vector for transmitting CHIKV throughout the world, including the central African region [59].

Detection of virus from male mosquitoes is also suggestive of TOT, apart from venereal transmission. As confirmed by a prior study, the infection rate of 11 % was obtained from virgin female *Ae. aegypti* cohabiting with the CHIKV-infected males [60]. This mechanism is one of the important modes that maintains the virus in nature. Additionally, the capacity of CHIKV to be vertically transmitted together with the characteristic of desiccation-resistant eggs of *Ae. aegypti* and *Ae. albopictus* mosquitoes likely combines to facilitate the survival of the virus during unfavorable inter-epidemic periods, which may increase the risk of CHIKV outbreak in the future.

## Conclusions

This study is the first report to demonstrate the number of successive generations of *Ae. aegypti* and *Ae. albopictus* mosquitoes reared in the laboratory that are able to transmit CHIKV IOL vertically in the laboratory. Moreover, our results indicate that *Ae. albopictus* appears to have a greater capacity for viral infection and transmission than does *Ae. aegypti*, which supports the evidence reporting *Ae. albopictus* as the major vector of this novel strain of CHIKV. Although *Ae. aegypti* showed less infection and transmission of CHIKV, it may play a crucial role as a competent vector and transmit the virus to humans in other parts of Thailand where it has a more intimate relationship with humans. However, the comparative study of transovarial transmission of CHIKV IOL in laboratory strains and natural field strains of the two mosquitoes are necessary to gain further insight and understanding of the observed differences between the two strains. Data on vertical transmission of the virus are beneficial to public health officials to assess the risk of CHIKV maintenance in nature, which is important for disease surveillance, vector control and the prevention of potential CHIK epidemics.
